# Risk of death and the economic accessibility at the dialysis therapy for the renal insufficient patients in Lubumbashi city, Democratic Republic of Congo

**DOI:** 10.11604/pamj.2014.19.61.3742

**Published:** 2014-09-23

**Authors:** Henri Mundongo Tshamba, Didier Van Caillie, Frank Nduu Nawej, Francis Mutach Kapend, Françoise Malonga Kaj, Grevisse Ditend Yav, Pascal Tshimwang Nawej

**Affiliations:** 1 Faculty of Medicine, University of Lubumbashi, Lubumbashi, Democratic Republic of Congo; 2 School of Management, Centre for Research on Corporate Performance, University of Liege, Liege, Belgium; 3 Centre Medical du Centre Ville, Lubumbashi, Democratic Republic of Congo; 4 Faculty of Economy and Management, University of Lubumbashi, Lubumbashi, Democratic Republic of Congo

**Keywords:** Dialysis, risk of death, economic accessibility, Lubumbashi, Democratic Republic of Congo

## Abstract

The last five years, Lubumbashi records the emergence centers of dialysis. We achieved this study to evaluate the risk factors of death for the renal insufficient patients and the economic accessibility to this peak therapy. A cross sectional study based on a random sample of 53 patients has been completed in 2012. The data is analyzed using the SPSS 19.0 software. A significance level of p < 0.05 and Confidence interval fixed to 95%. The Fischer exact test and the odds ratio have been used. The participation rate was 65.4%. The mean age was 49.49 ± 13.30 years old and 60.4% were aged > 50 years old. The sex ratio 0.3 women by men was noted. 83% of patients was private versus other category (p<0.05). 66% are renal insufficient chronic patients versus 34% of recent renal insufficient patients. 90% of patients were diabetic hypertensive. The patients’ monthly income declared was US$ 205 for 52.8% of patients, US $ 525 for 34% patients and US $ 750 for 13.2% of patients versus US $ 1, 270 monthly mean care cost. The deaths are associated statistically with an interruption of the treatment (χ^2^=9.30, p=0.0022, OR= 8.5) and with the irregularity of treatment (χ^2^=8.65, p=0.0032, OR=6). Africa in comparison with countries of other continents, to invest in advanced medical equipment is a salutary measure, but the majority of patients are not able to pay the costs of health care. Our results shown that, the dialysis became an ultimate recourse for the renal insufficient patients at Lubumbashi city but the economic accessibility remains a major obstacle. Consequently, it's important to subsidize the health care of these patients.

## Introduction

It's observed that the health consequence of chronic kidney disease is the renal failure. When renal function has deteriorated to a point when it is not possible to sustain life, the process is irreversible and the patient is considered to be in the end-stage renal disease [1]. Two methods of treatment are available for these patients: dialysis and renal transplantation. The development of these two methods has been an important advance of modern medicine, which has an impact on many renal patients [1].

In United States since 1996, around 335,014 patients in the depended on either dialysis or a kidney transplant to perform the function of their own failed kidneys [1, 2]. As well has, the health care is expensive and many life are saved. The study carried out by.‘. indicate that, the morbidity and mortality experienced by the treated end-stage renal disease population is substantially higher than for the all population [2]. Concerning the age of renal insufficient patients in the world, especially in the United States of America, some studies observed that the African-Americans people have develop end-stage renal failure at an earlier age than whites. For theses patients, their mean age at end-stage renal disease incidence was 55.8 years old compared with 62.2 for whites [2]. African-Americans people constitute almost 30 percent of prevalent end-stage renal disease patients, in spite they represent only 12.6 percent of the U.S. population [2]. The incidence of chronic kidney disease is enormous and its prevalence keeps increase [3]. It is highly important to highlight the burden of renal insufficient patients in developing countries in this century because the prevalence of kidney failure is increasing and their costs very high.

For example, the passage in 1972 for the Social Security Amendment P.L.92-603 instituted federally financed health care in the USA indicates that the cost of this program has far exceeded comparing to the original expectations. Medicare spending in 1996 was estimated to be $10.96 billion, and 12.5% increase from the $ 9.74 billion spent in 1995. At the same time, total end-stage renal disease spending by all payers was estimated to be $14.55 billion in 1996, up from $13.05 billion in 1995 (USRDS, 2010). Although there have been modest increases in the cost per patient, the driving force behind the growth in end-stage renal disease expenditures has been the increase in number of patients [1]. However, under some circumstances, the progression of renal disease can be slowed when a good follow up is observed. Three interventions have been shown effectiveness in some populations: good glycemic control for patients with diabetes mellitus, optimum blood pressure control for hyrtensive patients etc. [3]. The World Health Report 2002 and Global Burden of Disease project reports indicate that the kidney disease and urinary tract failure contribute to the global burden of renal failure. In this case, it caused approximately 850,000 deaths every year and 15,010,167 disability-adjusted life years. Globally, they represent the 12th cause of death and 17th cause of disability [4].

For example, recent studies oriented on patients with type 1 of diabetes mellitus have established that tight control of the level of blood sugar can reduce the development of proteinuria [3], and the use of angiotensin converting enzyme inhibitors can slow the progression of kidney disease [4]. There is substantial evidence that optimum blood pressure control is an important goal in the follow-up for all patients with proteinuria or chronic renal insufficiency [5, 6], and in patients with type 2 diabetes mellitus [7, 8]. The analysis of 2012 report of the Clinic CMDC, one dialysis center in Lubumbashi city reveals that 81 patients have been treated by dialysis among which 41 persons are insufficiency renal chronic and 40 persons have recent renal insufficiency. The passive indirect observation reported a large number of deaths that increases exponentially among renal insufficient patients. In light of these observations, it is necessary to consider the reasons for these findings. What is a profile of patients who discontinue treatment.

In addition, we detailed description of the death among the patients in this study. Finally, it's also important to analyze a level of economic access comparing to the costs of care with monthly income patients. A look at the fee schedule dialysis enough to realize the direct costs shown in this health care institution. Indeed, with a cost of US $ 317.50 per hemodialysis session in 2012. The dialysis care would likely be out of reach for the majority of patients in the Democratic Republic of the Congo, countries where much of the population lives under the poverty.

Furthermore, it's important to consider that if the frequency of hemodialysis patients in acute phase rarely exceeds half a dozen sessions. Chronic patients with end-stage disease requires three or more sessions per week, which causes high costs to patients and society. In the opinion of the experts, the causes of kidney failure are varied and the most important are hypertension and diabetes. In Nigeria, the situation is such that chronic kidney disease represents 8 to 10% of hospital admission [9–11]. The couple Hypertension and renal insufficiency is designated by the unromantic name “deadly duo”. For Menno T.et al. in 2008, Hypertension is present in 80% of chronic renal insufficient patients. In these patients, hypertension accelerates the deterioration of renal function and is the direct cause of renal insufficiency in a third of patients on dialysis [12].

Worldwide, the WHO estimates that one in three adults has high blood pressure and 40 to 50% of African adults are hypertensive. Which predisposing this category of patients to develop kidney insufficiency if the management of hypertension is not well realized. In the Democratic Republic of the Congo, the prevalence of hypertension among adults aged over 25 years old in 2008 was 38.5% in men; 33.3% among women which findings corroborate with some one indicating that n the sub-Saharan Africa the prevalence was 36.8% (34.0 - 39.7) [13]. The comorbidity hypertension and diabetes mellitus prevalence is increasing. The importance of these two diseases suggests that the level of risk of renal failure in diabetic hypertensive patients is theoretically higher. It's clear that the direct and indirect cost “Out of Pocket” in the context of patients who live in poverty around the world is both a source of impoverishment and a barrier to access to the health care. The cost of management of end-stage renal disease is prohibitive [14].

In developing countries where renal replacement therapy is available, but it is unaffordable by most patients. In Nigeria as in most other developing countries, there is not social security system or health insurance scheme to assist the patient and the burden is borne solely by the patient and relatives [3]. Health systems are confronted with this situation, should they all use the health insurance policy and risk sharing disease? To which hospital costs? These are reasons besides this study not only to document libraries and serve as a scale of costs for managers, charities and patients but also governments. Investigate the potential risk factors of death for renal insufficient patients can awaken the attention of physicians on the quality of care, sensitization the patients and their families about the importance of care and the role to be played by each actor to improving the quality of life for the renal insufficient patient.

## Methods

**Type and period of study:** this cross sectional study was achieved at the “Clinic CMDC“ Lubumbashi. This clinic is a specialized healthcare unit. The clinical is one of clinic specialized in hemodialysis. It operates since 2011 and has a technical platform that meets the requirements of medical technology and dialysis. 80% of the staff of the dialysis unit have received specific training in South Africa, through the partnership with Fresenius Medical care. Five equipment of dialysis is used in this medical center, which represents an important investment.

**Sampling:** a random sample of 53 patients (participation rate was 65.4%) treated by hemodialysis during the year 2012, and follow-up during one year. Free and informed consent of each participant in the study was obtained.

**Data collection and analysis:** the literature review and monthly reports of the medical center enables us to analyze patient records. The structured interview of patients or their relatives has enriched the data and clarify some aspects of the patient's life and medical care. Data were coded and analyzed using SPSS 19.0 software. A significance level of p <0.05 and Confidence interval fixed to 95%. The Fischer exact test and the odds ratio have been used.

## Results

**Type of renal insufficiency:** overall diabetic hypertensive patients have accounted for the majority of renal insufficient patients (90%). About the type of kidney failure, the results indicate 18 (34%) against acute renal failure 35 (66%) of patients in a state of chronic renal failure (Table 1).

**Table 1:** Baseline characteristics of 53 renal insufficient patients

Variables	Value Patients (n = 53)	Std Dev	P Value
Age, Mean years old (All sex)	49,49 (15-75)	13,30	
Sex ratio (Woman / Man)	15/53 (0.3)		< 0,05
Private patients	44 (83.0%)		< 0,05
Agent of the public administration	2 (3.8%)		
Agent of mining society	4 (7.5%)		
Agent of private enterprise	3 (5.7%)		
Monthly average income declared	129 (CI 95% : 105-525)		
Care cost per patient and by session of treatment (US $)	317,50		
Average month of treatment	2,8	2,7	
Average session of treatment by month	4,2	2.7	
Health Insurance			
Patients not insured	44 (83%)		< 0,05
Insured patients	9 (17%)		
Type of renal insufficient patients			
recent renal insufficiency	18 (34%)		0,042
Insufficiency renal chronic	35 (66%)		

**Age, sex and category of patients:** the average age of patients was 49.49 ± 13.30 years old and 60.4% were > aged 50 years old. The sex ratio was 0.3. A predominance of male patients is observed during this hospital study. 83% of patients was private versus other category (p<0.05) (Table 1).

**Risk factors of Death and lethality:** analysis of observed deaths indicates that the risk of death is increased by 6 for patients presented irregular of treatment (χ^2^= 8.65, p = 0.0032, OR = 6 (95% CI: 1.72-20.82)). Concerning this observation, irregularity is related to 77% of patients with limited financial resources, the stop treatment was a second risk factor of death for patients who were on dialysis (χ^2^= 9.30, p = 0.0022, OR = 8.5 (95% CI: 1.89-39.43). We have observed a significant difference in lethality between chronic renal insufficient patients compared to patients with renal insufficient in the acute phase (χ^2^= 5.14, p = 0.023, OR = 4.72 (95% CI: 1.15-19.25) (Table 2). When the number of dialysis session increases the proportion of deaths decreased (χ2= 6.33, p = 0.011, OR = 4.61 (95% CI: 1.34-15.78). The other sociodemographic and clinical factors did not show any association with the lethality observed in this study.

**Table 2:** Risk of death for 53 renal insufficient patients

		Death Value (n= 53)					
Variables		Yes	Not	Total	χ2	p	Decision
Number of dialysis sessions	≤ 5 sessions	20	5	25	6,796	0,033	S
	6-10 sessions	12	13	25			
	> 10 sessions	1	2	3			
Type of renal insufficient	recent renal insufficiency	15	3	18	5,14	0,023	S
	Insufficiency renal chronic	18	17	35			
Stop of treatment	Yes	32	10	42	9,3	0,0022	S
	Not	3	8	11			
Irregularity of treatment	Yes	22	5	27	8,65	0,0032	S
	Not	11	15	26			
Patients’ status	Private patients	28	16	44	8,071	0,045	S
	Agent of the public administration	2	0	2			
	Agent of mining society	3	1	4			
	Agent of private enterprise	0	3	3			

**Figure 1:**
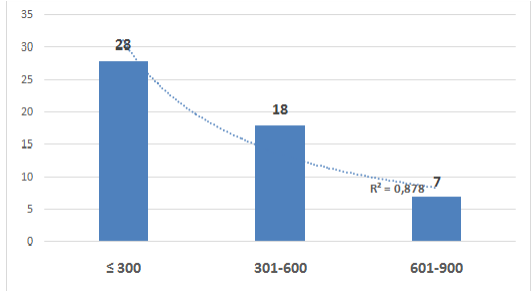
Patients’ monthly income declared

**Cost of health care:** according to the dialysis session the patient spends are US $ 317.50 direct cost (Table 1). The patients’ monthly average income declared was US $ 205 for 52.8% of patients, US $ 525 for 34% of patients and US $ 750 for 13.2% of patients versus US $ 1, 270 monthly mean cost care. These results indicate that the average monthly cost of US $ 1,270 is difficult to cover by patients from their own resources (Figure 1).

## Discussion

This study based of the risk of death and affordability for renal insufficient patients is an approach to public health and health economics in a discussion around the world. It shown that renal failure is largely a result of poor management of diabetes mellitus and hypertension or comorbidity hypertension and diabetes mellitus. Although it is useful to quantify the high costs of diabetes, it is also important to understand the underlying causes.

Our results indicate a rather alarming lethality around 62.2% of renal insufficient patients in a dialysis center at Lubumbashi city. It must indeed recognize that despite significant progress in therapy, the mortality in patients with acute renal failure remains high, estimated at around 50% [1, 2]. Several authors agree to highlight, using different fitting techniques, that there is a mortality directly related to acute renal failure [3, 4]. In a large study published, including more than 17,000 patients [15], using the technique of control cases had observed an increase in mortality from 38.5 to 62.8% when the acute renal failure was present.

These observations do not necessarily imply that acute renal failure is the cause of all of this difference in mortality as death and renal disease can both result from one or more third factors. But it's not so symmetrical, more conceivable that acute renal failure is quite independent of the high mortality [6]. Various pathophysiological hypotheses are possible to identify a common explanatory factor. The most obvious is probably the decrease in tissue perfusion due to a reduction in cardiac output that could explain both an important part of early mortality and renal impairment, since the kidney is physiologically very sensitive to reduction of blood flow. This hypothesis is also supported by the increased mortality accompanying the presence of acute renal failure in populations of patients with cardiovascular disease mortality shifts from less than 10% to over 65% when failure acute kidney was present [7]. About the type of kidney failure, the results indicate 18 (34%) against acute renal failure 35 (66%) of patients in a state of chronic renal failure. We observed a significant difference in lethality between chronic renal impairment compared to patients with renal insufficiency in the acute phase (p = 0.023), but also when the number of dialysis increases the proportion of deaths decreased (p = 0.011). These findings support the need of dialysis therapy for renal insufficient patients.

Besides cardiovascular complications, deaths among dialysis patients are due to infections, especially in case of long-term catheter, which multiplies by 8 time the risk of severe sepsis. Death may also occur in an array of cachexia with malnutrition promoting complications, including cardiovascular disease. Clinical investigations, hemodynamic and nutrition are important for patients undergoing dialysis sessions. The correction of anemia in the renal insufficient patient meets good practice guidelines that are based on four principles. The first and the second principle are the use of erythropoiesis-stimulating agents (ESA) and the prescription of iron as adjuvant factors. But optimizing dialysis program also requires the correction of anemia, framed by European guidelines (European Best Practice Guidelines EBPG), from the French Agency for the Safety of Health Products (AFSSAPS) of National Kidney Foundation (the Kidney Disease Outcomes Quality Initiative NKF KDOQI) and the International initiative KDIGO (Kidney Disease Improving Global Outcomes). Good clinical practice in hemodialysis recommend that anemia correction is achieved in the first time to six months of treatment [16].

In addition, the average age of patients was 49.49 ± 13.30 years old and 60.4% were aged > 50 years old. The sex ratio of 15/53 (0.3) was noted, and a predominance of male patients is observed. Age is a fairly documented in renal failure factor. The age of individuals correspond to those of their arteries state, which explains the particularly high cardiovascular risk. There is a continuum in the development of these vascular structural, functional and hemodynamic lesions, real gear that starts when chronic renal failure begins [7, 12, 17]. About the status of the patients, we found that 83% patients was private versus other category (p <0.05). The majority of the private patients lack the financial resources to pay their healthcare expenses. The results indicate that the risk of death is increased 6 time for the patients with irregular treatment (p = 0.0032). This irregularity is related to 77% of patients with limited financial resources is observed and the discontinuation resulting for it is a second risk of death for patients who were on dialysis (p = 0.0022). We observed a significant difference in lethality between chronic renal insufficiency patients compared to patients with renal insufficiency in the acute phase (p = 0.023). When the number of dialysis session increases the proportion of deaths decreased (p = 0.011), demonstrating unequivocally the need for dialysis therapy for these patients. Many studies show that the risk of death is high for more chronic renal failure patients. The risk of death from all causes is multiplied 6 time for patients in end state [16]. Otherwise, by dialysis, the patient pays US $ 317.50 direct cost. But, the patients’ average monthly income declared was US $ 205 for 52.8% of patients, US $ 525 for 34% and US $ 750 for 13.2% of patients versus US $ 1,270 monthly mean cost care. The results indicate that the average monthly cost of US $ 1,270 is difficult to cover by patients from their own resources.

In the study carried out by Philip Clarke et al. (2006), the results indicate that the overall average hospital cost per year for diabetes patients was $ 3676 (SD, 7756) compared to $ 2670 (SD, 6045) for control patients. Forty percent of the $ 1,005 (95% CI: 927-1084), were excess hospital costs due to higher disease-specific costs, reflecting greater intensity of treatment, with the remainder due to the higher frequency of hospitalization. It was found that although treatment costs for a new comorbidity peaked in the first year for both groups, thesis continued higher costs over subsequent years for people with diabetes [18]. In recent years, considerable research has been undertaken using administrative data to understand the relative health care costs of people with and without diabetes. For all, the greatest proportion of costs generally attributed to hospital care [19].

The excess hospital costs, that is the additional cost of treating diabetes patients compared with matched control patients, are a combination of two factors. First, it is well to known that the diabetes patients are higher risk of cardiovascular disease and many other complications that result to higher frequency of conditions that require hospitalization for treatment [20, 21]. In Africa, at Lubumbashi city and other parts around the world, family solidarity and social dynamics participation around the patient allows the patient to pay the health care cost. For chronic diseases such as hypertension, diabetes mellitus and renal failure which is the complication, the cost would be too great outside hospital because the patients must monitor their blood glucose and renal clearance and often consult physicians when necessary and they must buy the drugs daily.

## Conclusion

The results of this study indicate the need for further investigations on the slopes of successful dialysis, the risk factors for death for renal insufficient patients and the procedures for grant expenditures that support. The irregularity of dialysis therapy was significant risk factor of death. The hospital costs are very high compared to the monthly income. Our results shown that, the dialysis became an ultimate recourse for the renal insufficient patients at Lubumbashi city but the economic accessibility remains a major obstacle. Consequently, it's important to subsidize the health care of these patients.
